# *PLD3* Rare Variants Identified in Late-Onset Alzheimer’s Disease Affect Amyloid-β Levels in Cellular Model

**DOI:** 10.3389/fnins.2019.00116

**Published:** 2019-02-14

**Authors:** Mengshan Tan, Jieqiong Li, Fangchen Ma, Xing Zhang, Qingfei Zhao, Xipeng Cao

**Affiliations:** ^1^Department of Neurology, Qingdao Municipal Hospital, Qingdao University, Qingdao, China; ^2^Department of Neurology, Qingdao Municipal Hospital, Weifang Medical University, Qingdao, China; ^3^Department of Neurology, Qingdao Municipal Hospital, Dalian Medical University, Dalian, China; ^4^Clinical Research Center, Qingdao Municipal Hospital, Qingdao University, Qingdao, China

**Keywords:** Alzheimer’s disease, amyloid-β, PLD3, variant, pathogenesis

## Abstract

Next-generation sequencing studies have reported that rare variants in *PLD3* were associated with increased risk of late-onset Alzheimer’s disease (LOAD) in European cohorts. The association has been replicated in a Han Chinese cohort, two rare variants p.I163M in exon7 and p.R356H in exon11 of *PLD3* were found to be associated with LOAD risk. Whether these variants have deleterious effects on protein function, and the underlying mechanisms by which they influence LOAD pathogenesis are unknown. Our results are the first to validate the hypothesis that these variants could lead to reduced PLD3 activity and affect amyloid-β levels in cellular model of AD, possibly via autophagy-dependent mTOR signaling pathway, indicating that PLD3 may represent a new therapeutic target for AD.

## Introduction

Recent advances in next-generation sequencing technology have made it possible to identify novel functional variants with large effect sizes associated with Alzheimer’s disease (AD) risk. Studies showed that rare variants in *phospholipase D3* (*PLD3*) gene were associated with late-onset AD (LOAD) in European cohorts (Cruchaga et al., 2014; Schulte et al., 2015), and a coding variant p.V232M in *PLD3* increased AD risk by twofold (Cruchaga et al., 2014). However, some replication studies failed to find the association of p.V232M variant with AD risk (Cacace et al., 2015; Heilmann et al., 2015; Hooli et al., 2015; Lambert et al., 2015), and its contribution to the phenotype has not been confirmed according to OMIM database. Currently, the association of *PLD3* with LOAD has been replicated in Han Chinese cohort for the first time by our research group, and two rare variants p.I163M and p.R356H in exon regions of *PLD3* are found to increase LOAD risk (Tan et al., 2018). Although p.R356H, also identified in a European cohort previously (Schulte et al., 2015), was present only in our LOAD patients, this association of p.R356H with LOAD risk did not reach statistical significance after Bonferroni correction, which might be due to its rarity (Tan et al., 2018). Considering their probably deleterious effects on PLD3 functions based on Polyphen-2 and SIFT scores (Tan et al., 2018), and the evidence that variants in *PLD3* were associated with amyloid pathology and cognitive decline (Wang et al., 2015, 2016; Lin et al., 2017; Engelman et al., 2018), we need to assess the functional consequence of these variants and investigate the possible mechanisms by which they influence AD pathogenesis.

Phospholipase D3 is highly expressed in hippocampus and cortex, regions more vulnerable to AD pathology (Cruchaga et al., 2014). PLD3 mRNA and protein expression are decreased in LOAD patients brain (Cruchaga et al., 2014; Satoh et al., 2014). Notably, PLD3 accumulates in neuritic plaques (Satoh et al., 2014), and functions in regulating the processing of amyloid-beta (Aβ) precursor protein (APP; Cruchaga et al., 2014; Guimas Almeida et al., 2018). Further studies showed PLD3 colocalized with APP in endosomes and loss of PLD3 function resulted in increased processing of APP to Aβ (Mukadam et al., 2018). It should, however, be noted that genetic knockout of *PLD3* in mice did not result in altered APP processing or increased Aβ levels (Fazzari et al., 2017). Considering the PLD family, which includes PLD1 and PLD2, both involved in endocytic trafficking and APP processing, might have impacts on the results in animal models of AD (Oliveira and Di Paolo, 2010), we choose the cellular model, HEK293 cells expressing the Swedish mutant of APP695 (HEK293-APP695), for the current study.

## Materials and Methods

### Plasmids

Full-length cDNA sequence of *PLD3* was obtained from National Center for Biotechnology Information (NCBI). The *PLD3* p.I163M or p.R356H variant was introduced into the pcDNA3.1-EGFP expression vector encoding human wild-type (WT) *PLD3* by Keygen Biotech. Co. Ltd. (Nanjing, China) using the site-directed mutation method (Stratagene, La Jolla, CA, United States). As a result, all the *PLD3* encoding expression vectors were EGFP-tagged. The plasmid sequences were verified by Sanger sequencing.

### Cell Culture, Transfection, and Treatment

HEK293 cells stably expressing the Swedish mutant of APP695 (HEK293-APP695) were a generous gift from Dr. Teng Jiang (Department of Neurology, Nanjing First Hospital, Nanjing Medical University, Nanjing, China; Jiang et al., 2014). Cells were grown in Dulbecco’s modified Eagle medium supplemented with 10% fetal bovine serum and 1% penicillin-streptomycin in a 37°C incubator with 5% CO_2_. Cells were transfected with an empty EGFP vector or EGFP-*PLD3* WT, EGFP-*PLD3*-I163M, and EGFP-*PLD3*-R356H expressing plasmids. Transfections were performed using polyethylenimine (Polysciences) according to the manufacturer’s instructions. Cells were cultured 48 h post-transfection. Transfection efficiency was tested by real-time quantitative PCR method.

Rapamycin (RAPA; Melone Pharmaceutical Co., Ltd., Dalian, China) was dissolved in dimethyl sulfoxide (DMSO, Sigma–Aldrich). For inhibition of mTOR to enhance autophagy experiments, the cells were pretreated in the presence or absence of 0.2 μM RAPA for 24 h prior to plasmids transfection as described above for 48 h.

### Real-Time Quantitative PCR

Total RNA was extracted from the cells using the Trizol reagent (Invitrogen) according to the manufacturer’s protocol. Samples were reverse-transcribed into cDNA using the Prime-Script one step RT reagent Kit (TaKaRa, Madison, WI, United States). Synthesized cDNA was used in real-time PCR experiments using the SYBR Premix Ex Taq (TaKaRa), and then analyzed with CFX96 Real-Time PCR detection system (Bio-Rad). All reactions were run in triplicates and the mean values are used. The relative expression of each mRNA was calculated using the comparative 2^-ΔΔCt^ method and was normalized against glyceraldehyde-3-phosphate dehydrogenase (GAPDH). cDNA amplification was performed by using the following primers: 5′-ATGAAGCCCAAACTGATGTACC-3′ (forward) and 5′-AAGTCCCCGTATTCCCATAGAA-3′ (reverse) for PLD3. 5′-AGGCCGGTGCTGAGTATGTC-3′ (forward) and 5′-TGCCTGCTTCACCACCTTCT-3′ (reverse) for GAPDH.

### MTT Assay for Cell Viability

Cell viability was determined using a commercial MTT-based cytotoxicology test kit (Sigma–Aldrich, United States), which detects viable cells colorimetrically based on the detection of the purple formazan compound produced by viable cells. Cells were seeded in 96-well plates. After removing the supernatant of each well and washing twice by PBS, 20 μl of MTT solution (5 mg/ml in PBS) was added and the culture was further incubated for 4 h. Then, the culture medium was replaced with 100 μl of MTT solubilization solution. The absorbance at 570 nm was measured using a microplate reader (Bio-RAD, United States). All treated samples and controls were tested in triplicate. Incidentally, the cell viability between EGFP-transfected and non-transfected cells did not differ, excluding an effect of transfection on cell viability.

### Phospholipase D Activity

Phospholipase D3 activity was measured using a colorimetric assay [Phospholipase D (PLD) Activity Colorimetric Assay Kit; BioVision, San Francisco, United States] following manufacturer’s instructions.

### Western Blot Analysis

For western blotting, cells were lyzed in extraction buffer (Beyotime Inc., China) containing complete protease inhibitor cocktail (Roche). The protein concentrations were determined using the BCA protein assay kit (Beyotime Inc., China). Different samples with an equal amount of protein were separated on 10–15% SDS polyacrylamide gels, and transferred to PVDF membranes. The membranes were blocked with non-fat milk and incubated at 4°C overnight, with the primary antibodies against PLD3 (1:200, #HPA012800; Sigma–Aldrich, United States), APP-FL (1:1000, #2452; Cell Signaling Technology, United States), BACE (1:1000, #5606; Cell Signaling Technology, United States), NEP (1:500, sc-46656; Santa Cruz Biotechnologies, United States), IDE (1:500, sc-27265; Santa Cruz Biotechnologies, United States), LC3 (1:1000, #4108; Cell Signaling Technology, United States), p62 (1:1000, #397749; Cell Signaling Technology, United States), phospho-mTOR (Ser2448, 1:1000, #2971; Cell Signaling Technology, United States), mTOR (1:1000, #2972; Cell Signaling Technology, United States), phospho-p70 S6K (Thr389, 1:1000, #97596; Cell Signaling Technology, United States), p70 S6K (1:1000, #9202; Cell Signaling Technology, United States), and β-actin (1:500, sc-47778; Santa Cruz Biotechnology, United States). After rinsing, the membranes were appropriately incubated with horseradish peroxidase (HRP)-conjugated suitable secondary antibodies (1:5000; Zhongshan Inc., China) for 2 h at room temperature. After washing, protein bands were detected with a chemiluminescent HRP substrate (Thermo Scientific Inc., United States) for 5 min at room temperature and exposed to X-ray film (Fujifilm Inc., Japan). The signal intensity was analyzed using Quantity One software 4.6.2 (Bio-Rad, United States) and normalized to the loading control β-actin.

### ELISA for Aβ Quantification

To evaluate the intracellular Aβ_42_ in HEK293/APPswe cells, the cell lysates were collected. The concentrations of Aβ_42_ were then detected by a specific ELISA kit (Invitrogen, United States). The change in absorbance in each well at 450 nm was detected with a spectrophotometer (GE Healthcare).

### Statistical Analysis

Statistical analysis was conducted by SPSS software 13.0. Independent sample *t*-test or one-way ANOVA followed by Tukey’s HSD (honestly significant difference) *post hoc* test were used to analyze differences among groups. All data are expressed as mean ± SEM. *P* < 0.05 was considered statistically significant.

## Results

### The *PLD3* Mutations Lead to Reduced Phospholipase Activity

**FIGURE 1:**
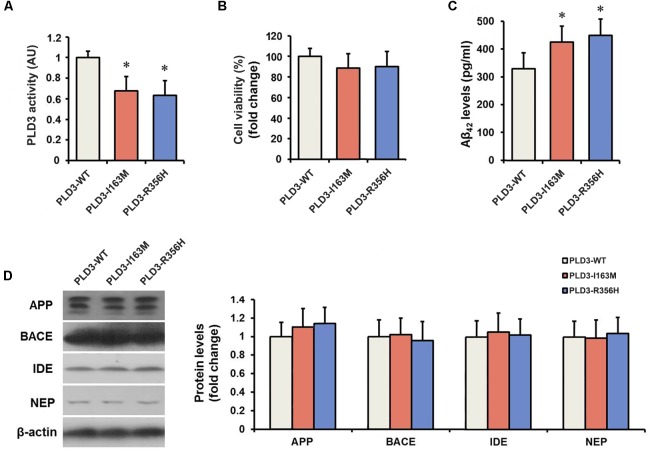
The *PLD3* mutations lead to reduced phospholipase activity and increased Aβ levels in HEK293-APP695 cells. **(A)** Quantification of PLD3 activity in cell lysates. Cells were transfected with PLD3-WT, PLD3-I163M, or PLD3-R356H for 48 h, and the activity was measured using a commercial available colorimetric assay. **(B)** Cell viability was determined by MTT assay and shown as a percentage of surviving cells. **(C)** The intracellular Aβ_42_ levels were evaluated by ELISA. **(D)** The protein levels of APP, β-secretase (BACE), insulin-degrading enzyme (IDE), and neprilysin (NEP) were measured by western blotting, and quantified by densitometric measurement. β-actin was used as loading control. ^∗^*P* < 0.05 versus cells treated with PLD3-WT group. The plots represent the mean ± SEM. All data shown are representative of three independent experiments, performed in triplicate.

We determined the PLD activity of PLD3 in transfected cells. PLD3-I163M and PLD3-R356H exhibited significantly reduced activity compared to PLD3-WT transfected cells (Figure 1A), validating the damaging effects of the p.I163M and p.R356H variants. No significant differences were observed in mRNA or protein levels of PLD3 among the PLD3-I163M, PLD3-R356H, and PLD3-WT groups (Supplementary Figure S1). Cell viability was then assessed by MTT assay. As shown in Figure 1B, the percentage of surviving cells has no significant difference in PLD3-I163M and PLD3-R356H transfected cells compared with PLD3-WT transfected cells.

### The *PLD3* Mutations Lead to Increased Aβ Levels

As revealed by Figure 1C, PLD3-I163M and PLD3-R356H significantly increase intracellular Aβ_42_ levels in transfected cells. It is worth noting that the levels of β-secretase (BACE) stayed unchanged, although the APP levels showed an increasing trend in PLD3-I163M and PLD3-R356H transfected cells. Meanwhile, we found that the amounts of insulin-degrading enzyme (IDE) and neprilysin (NEP), the two major Aβ-degrading enzymes, were unaltered (Figure 1D), indicating these mutations induced the increasing of Aβ was not accomplished by affecting its enzymatic degradation.

### The PLD3 Mutations Affect Aβ Levels via mTOR Pathway

**FIGURE 2:**
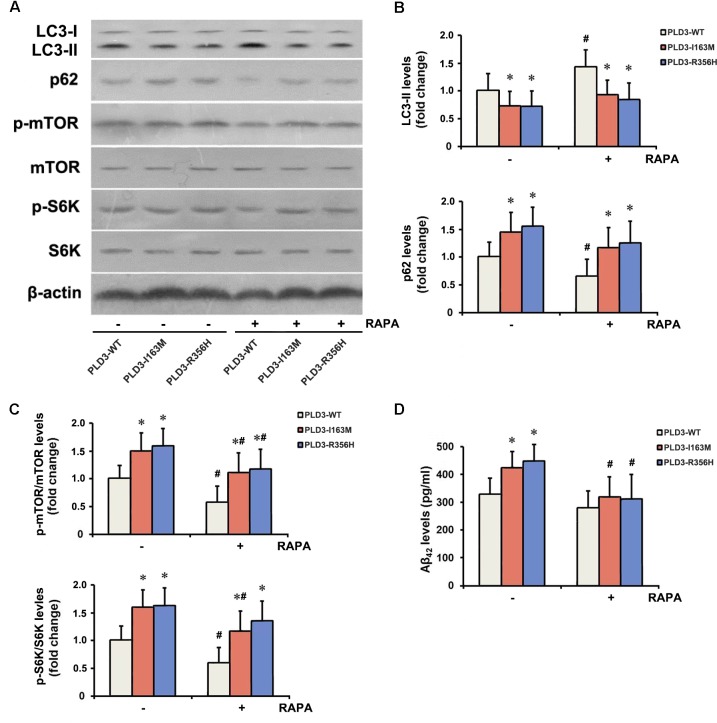
The *PLD3* mutations affect Aβ levels via autophagy-dependent mTOR pathway. HEK293-APP695 cells were pretreated in the presence or absence of 0.2 μM RAPA for 24 h prior to transfection with PLD3-WT, PLD3-I163M, or PLD3-R356H for 48 h. **(A)** The protein levels of LC3-II and p62, as well as the ratios of p-mTOR/total mTOR and p-S6K/total S6K were detected by western blotting. **(B)** Quantification of LC3-II and p62 levels. **(C)** Quantification of ratios of p-mTOR/total mTOR and p-S6K/total S6K. **(D)** The Aβ_42_ levels were evaluated by ELISA. ^∗^*P* < 0.05 versus cells treated with PLD3-WT group. ^#^*P* < 0.05 versus cells pretreated in the absence of 0.2 μM RAPA prior to plasmids transfection. The plots represent the mean ± SEM. All data shown are representative of three independent experiments, performed in triplicate.

To examine whether *PLD3* mutations were involved in the regulation of autophagy in cellular model, western blotting was performed to detect the expression levels of the autophagy-associated proteins LC3-II and p62. These *PLD3* mutations reduced the level of LC3-II and increased the level of p62 protein (Figure 2A,B), suggesting autophagic activity was remarkably reduced. Autophagy is controlled by several kinases including mTOR, a negative regulator of autophagy. Further study showed that these *PLD3* mutations increased the phosphorylation of mTOR and S6K (a downstream target of mTOR; Figure 2A,C), suggesting that mTOR was markedly activated. We further investigate whether mTOR signaling was involved Aβ accumulation induced by PLD3 mutations. As shown in Figure 2A–C, the notion that the inhibition of mTOR kinase by RAPA could induce autophagy was confirmed. As demonstrated by Figure 2D, in the presence of RAPA, these *PLD3* mutations failed to increase Aβ_42_ levels in HEK293-APP695 cells.

## Discussion

In the present study, we show that *PLD3* rare variants p.I163M and p.R356H identified in LOAD could lead to reduced PLD3 activity and affect Aβ levels in cellular model of AD, via autophagy-dependent mTOR pathway. These findings indicate that PLD3 may represent a new therapeutic target for AD in cellular model.

According to the amyloid hypothesis, accumulation of Aβ is the critical pathogenic event in AD (Hardy and Selkoe, 2002). Tremendous efforts have been made to develop therapies targeting Aβ production. PLD3 accumulates in neuritic plaques (Satoh et al., 2014), and functions in regulating the processing of APP (Cruchaga et al., 2014; Guimas Almeida et al., 2018). PLD3 loss-of-function increased secretion of Aβ_42_ and Aβ_40_ (Mukadam et al., 2018). However, this result was not replicated in PLD3 knockout mice (Fazzari et al., 2017). Recently, accumulating evidence suggested a crucial role of impaired Aβ clearance rather than its overproduction in AD pathogenesis (Mawuenyega et al., 2010). Because overexpression of Aβ is needed to evaluate the influence of its levels induced by the mutations, in our current study, we used HEK293-APP695 cells with high Aβ expression which may facilitate the assays (Jiang et al., 2014).

Phospholipase D3 is a membrane-associated protein of the PLD family, involved in endocytic trafficking (Donaldson, 2009). Moreover, PLD, which catalyzes the hydrolysis of phosphatidylcholine to phosphatidic acid (PA), has been established as a key upstream component in the mitogenic mTOR pathway (Huang and Frohman, 2007; Valli et al., 2015). Numerous data have provided sufficient evidence for the relationship between the mTOR signaling pathway and AD. Alteration of mTOR signaling occurs early in the progression of AD (Tramutola et al., 2015). Application of mTOR inhibitors provides an innovative therapeutic strategy for AD (Wang et al., 2014). Our current study shows that variants p.I163M and p.R356H in exon regions of *PLD3* identified in LOAD affect Aβ clearance, possibly via activated mTOR pathway. These findings indicate PLD3 as a new player in regulating autophagy, and manipulation of PLD3 is likely to affect the autophagic clearance of Aβ in AD pathogenesis.

Next-generation sequencing studies identified p.V232M in exon 7 of *PLD3* as the potentially functional rare variant with causal effect on the development of LOAD in European cohorts (Cruchaga et al., 2014). Furthermore, two rare variants p.I163M and p.R356H in exon regions of *PLD3* were found to increase LOAD risk in a Han Chinese cohort (Tan et al., 2018). The minor-allele frequency of p.I163M in our Han Chinese population (0.22%) is very similar that in East Asian population in the gnomAD database (0.28%). Remarkably, p.I163M has a relatively higher frequency in East Asian population than in other populations, indicating the importance and necessity of re-sequencing the susceptible gene in different populations. In addition, we identified a rare variant p.R356H that was present only in patients with LOAD, but was not associated with LOAD risk after Bonferroni correction, which might be due to its rarity (Schulte et al., 2015; Tan et al., 2018). This result requires further confirmation in Han Chinese by future studies with larger samples.

Phospholipase D3 protein consists of a transmembrane domain and two PLD phosphodiesterase domains with two copies of the catalytically essential HKD motif (Supplementary Figure S2). The p.I163M and p.R356H variants located in the phosphodiesterase domain, which is similar with the p.V232M variant, and have probably deleterious impacts on PLD3 functions based on Polyphen-2 and SIFT scores (Tan et al., 2018). To assess the functional consequences of these variants, we detect the PLD3 activity and amyloid-β levels in cellular model, validating the damaging effect of the p.I163M and p.R356H variants.

## Conclusion

Our current study provides the evidence that *PLD3* rare variants p.I163M and p.R356H identified in LOAD could lead to reduced PLD3 activity and affect Aβ levels in cellular model of AD, via autophagy-dependent mTOR pathway. These findings indicate that PLD3 may represent a new therapeutic target for AD, and manipulation of PLD3 is likely to affect the clearance of Aβ in AD pathogenesis.

## Author Contributions

MT designed the study, analyzed the data, and drafted the paper. JL, FM, XZ, and QZ carried out the experiments and analyzed the data. XC analyzed the data and checked the language expression. The final version was approved for submission by all listed authors.

## Conflict of Interest Statement

The authors declare that the research was conducted in the absence of any commercial or financial relationships that could be construed as a potential conflict of interest.

## Supplementary Material

FIGURE S1 Real-time quantitative PCR and western blotting analyses of PLD3 levels in HEK293-APP695 cells 48 h post-transfection. **(a)** Real-time PCR was used to quantify PLD3 mRNA and standardized using GADPH mRNA as a reference. **(b)** The PLD3 protein levels were measured by western blotting, and quantified by densitometric measurement. β-actin was used as loading control. The plots represent the mean ± SEM. All data shown are representative of three independent experiments, performed in triplicate.

FIGURE S2 Schematic representation of *PLD3* and the relative position of the *PLD3* variants. *PLD3* has two PLD phosphodiesterase domains, which contain an HKD signature motif [H-X-K-X(4)-D-X(6)-G-T-X-N, where X represents any amino acid residue]. The position of the p.I163M and p.R356H variants in *PLD3* is shown.
