# Supplementary figures and images for: PLD3 Rare Variants Identified in Late-Onset Alzheimer’s Disease Affect Amyloid-β Levels in Cellular Model
Source: Front Neurosci. 2019 Feb 14;13:116. doi: 10.3389/fnins.2019.00116 (PMC6382672; doi:10.3389/fnins.2019.00116)

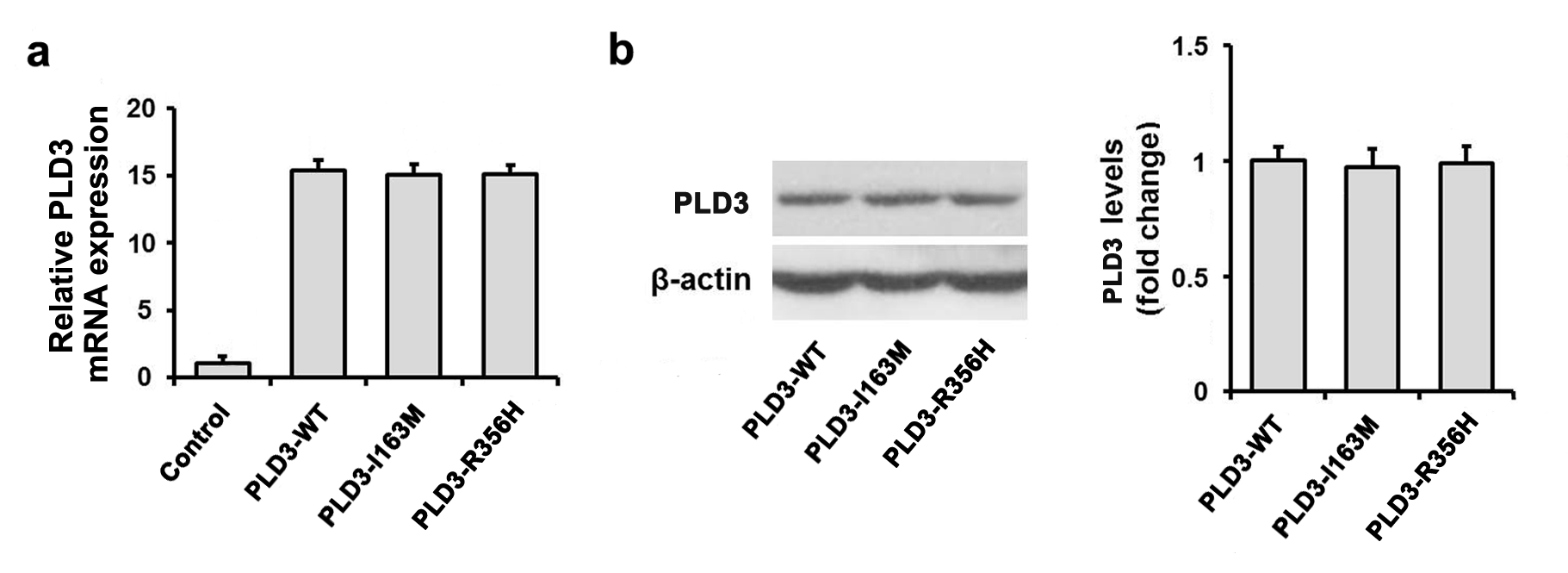

Supplement: FIGURE S1 — Real-time quantitative PCR and western blotting analyses of PLD3 levels in HEK293-APP695 cells 48 h post-transfection. (a) Real-time PCR was used to quantify PLD3 mRNA and standardized using GADPH mRNA as a reference. (b) The PLD3 protein levels were measured by western blotting, and quantified by densitometric measurement. β-actin was used as loading control. The plots represent the mean ± SEM. All data shown are representative of three independent experiments, performed in triplicate. [file Image_1.TIF]

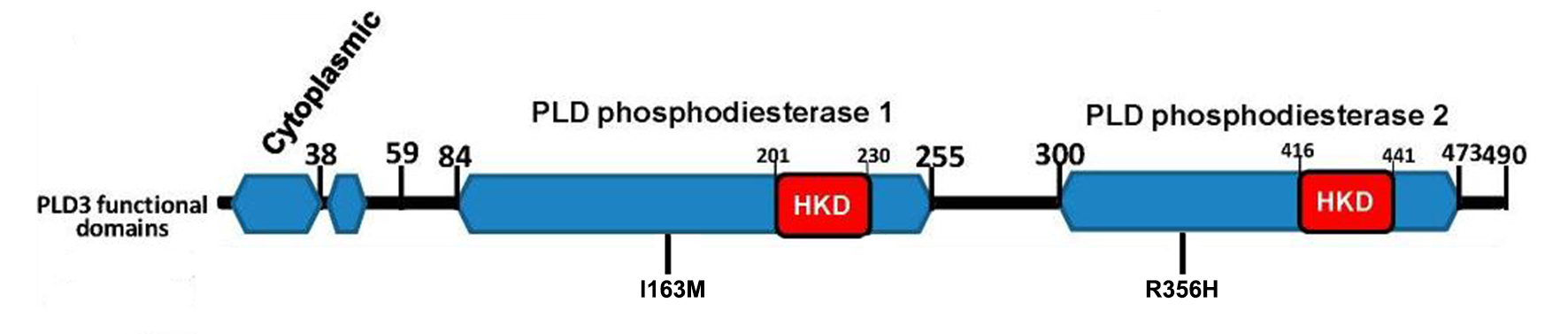

Supplement: FIGURE S2 — Schematic representation of PLD3 and the relative position of the PLD3 variants. PLD3 has two PLD phosphodiesterase domains, which contain an HKD signature motif [H-X-K-X(4)-D-X(6)-G-T-X-N, where X represents any amino acid residue]. The position of the p.I163M and p.R356H variants in PLD3 is shown. [file Image_2.TIF]
